# Validation of Candidate Host Cell Entry Factors for Bovine Herpes Virus Type-1 Based on a Genome-Wide CRISPR Knockout Screen

**DOI:** 10.3390/v16020297

**Published:** 2024-02-15

**Authors:** Wenfang Spring Tan, Enguang Rong, Inga Dry, Simon Lillico, Andy Law, Paul Digard, Bruce Whitelaw, Robert G. Dalziel

**Affiliations:** 1 Division of Infection and Immunity, the Roslin Institute, Easter Bush Campus, University of Edinburgh, Edinburgh EH259RG, UKinga.dry@roslin.ed.ac.uk (I.D.); paul.digard@roslin.ed.ac.uk (P.D.); robert.dalziel@ed.ac.uk (R.G.D.); 2 Division of Functional Genetics and Development, the Roslin Institute, Easter Bush Campus, University of Edinburgh, Edinburgh EH259RG, UK; simon.lillico@roslin.ed.ac.uk (S.L.); bruce.whitelaw@roslin.ed.ac.uk (B.W.); 3 Centre for Tropical Livestock Genetics and Health, the Roslin Institute, Easter Bush Campus, University of Edinburgh, Edinburgh EH259RG, UK; 4 Division of Genetics and Genomics, the Roslin Institute, Easter Bush Campus, University of Edinburgh, Edinburgh EH259RG, UK; andy.law@roslin.ed.ac.uk

**Keywords:** CRISPR/Cas9, BoHV-1, cell entry, receptors, heparan Sulfate, Golgi apparatus, COG complex

## Abstract

To identify host factors that affect Bovine Herpes Virus Type 1 (BoHV-1) infection we previously applied a genome wide CRISPR knockout screen targeting all bovine protein coding genes. By doing so we compiled a list of both pro-viral and anti-viral proteins involved in BoHV-1 replication. Here we provide further analysis of those that are potentially involved in viral entry into the host cell. We first generated single cell knockout clones deficient in some of the candidate genes for validation. We provide evidence that Polio Virus Receptor-related protein (PVRL2) serves as a receptor for BoHV-1, mediating more efficient entry than the previously identified Polio Virus Receptor (PVR). By knocking out two enzymes that catalyze HSPG chain elongation, HST2ST1 and GLCE, we further demonstrate the significance of HSPG in BoHV-1 entry. Another intriguing cluster of candidate genes, COG1, COG2 and COG4-7 encode six subunits of the Conserved Oligomeric Golgi (COG) complex. MDBK cells lacking COG6 produced fewer but bigger plaques compared to control cells, suggesting more efficient release of newly produced virions from these COG6 knockout cells, due to impaired HSPG biosynthesis. We further observed that viruses produced by the COG6 knockout cells consist of protein(s) with reduced N-glycosylation, potentially explaining their lower infectivity. To facilitate candidate validation, we also detailed a one-step multiplex CRISPR interference (CRISPRi) system, an orthogonal method to KO that enables quick and simultaneous deployment of three CRISPRs for efficient gene inactivation. Using CRISPR3i, we verified eight candidates that have been implicated in the synthesis of surface heparan sulfate proteoglycans (HSPGs). In summary, our experiments confirmed the two receptors PVR and PVRL2 for BoHV-1 entry into the host cell and other factors that affect this process, likely through the direct or indirect roles they play during HSPG synthesis and glycosylation of viral proteins.

## 1. Introduction

Bovine Herpes Virus Type 1 (BoHV-1) is a widespread virus in farmed cattle. Acute infection or reactivation from latency causes severe syndromes of the respiratory and reproductive systems, transient immunosuppression and co-infection with other microbes, leading to substantial economic loss annually to cattle industries worldwide [1,2,3].

Like other alpha herpesviruses, BoHV-1 is an enveloped large double strand DNA virus that enters the host cell via receptor-mediated membrane fusion either on the cell surface or within endosomes [4]. BoHV-1 entry is initiated by interactions between cellular surface molecules and viral envelope glycoproteins gB, gC, and gD [2,5,6]. Although not required for virus replication, gC plays a major role in attachment by interacting with cell surface HSPG [7,8], thereby bringing gB and gD into proximity with their receptors [2,9,10,11,12,13]. Specifically, gD binds to cellular receptors nectin-1 (PVRL1) and poliovirus receptor (PVR) [14,15,16], while gB or a gH/gL complex may interact with putative alphaherpesvirus gB-receptor PILRα, resulting in fusion of the viral envelope with the cell membrane [17,18,19,20,21,22]. Previous studies found that human cell surface protein PVRL2 can mediate efficient entry of Herpes Simplex Virus 2 and mutant strains of Herpes Simplex Virus 1 (HSV-1) but not wild-type HSV-1 or BoHV-1 into Chinese Hamster Ovary (CHO) cells [15,23,24]. Perhaps due to this negative outcome, the possibility of bovine PVRL2 serving as a receptor for BoHV-1 entry into cow cells has never been further explored.

Ubiquitously expressed by most mammalian cell types, HSPG is anchored to the plasma membrane or extracellular matrix. The transmembrane core protein of HSPG is covalently linked to chains of glycosaminoglycan (GAG) formed by unbranched sulfated polysaccharides, also known as heparan sulfates (HS) [25]. Other GAGs such as chondroitin sulfates (CS) and dermatan sulfates (DS) can also be attached to these core proteins [26]. The negatively charged GAG chains interact electrostatically with the basic residues of either the glycoproteins of enveloped viruses or the capsid proteins of non-enveloped viruses. A broad range of viruses, including Human Immunodeficiency Virus Type 1, Respiratory Syncytial Virus (RSV), Hepatitis E virus (HEV), HSV-1, Foot and Mouth Disease Virus [27] and BoHV-1 [7,8], overcome Brownian motion by exploiting such weak interactions and thereby increase their concentration at the host cell surface, boosting the probability of binding to more specific entry receptors for internalization [25]. Although with limited documentation, HSPG might even serve as a direct entry receptor for some of these viruses (HSV-1, HEV, and RSV) [28,29,30], while other GAGs have also been implicated in viral entry into host cells [31], e.g., CS binding by HSV-1 [32,33].

Like many other glycosylation and sulfation processes, the biosynthesis of HSPG is a multi-step reaction that takes place in the Golgi network. To initiate the process, biosynthetic precursors including 3′-phosphoadenosine-5′-phosphosulfate (PAPS) and UDP-sugars are transported into the Golgi lumen from the cytosol to serve as substrates [34]. Once the linkage region is attached to a serine residue in the core protein, the exostosins (EXT1, EXT2, EXTL1-3) [27,35] add alternating units of N-Acetylglucosamine (GlcNAc) and glucuronic acid (GlcA) to the growing chain, followed by a series of sulfation and epimerization modifications to the units [26]. Upon completion, the core proteins decorated with these HS chains are transported towards the plasma membrane for exocytosis. The proper intracellular distribution of these exostosins is crucial for successful completion of HS biosynthesis, and this is maintained by retrograde vesicle traffic between Golgi cisternae [36]. Vesicle tethering at the Golgi apparatus prior to fusion is mediated by Rab GTPase-coordinated interactions between the coiled-coil golgin tethers and the multi-subunit tethering complex known as the conserved oligomeric Golgi (COG) complex [36]. The COG complex is crucial for multiple aspects of the Golgi apparatus biology, including maintenance of its homeostasis, intracellular trafficking and protein glycosylation [37]. The significance of vesicle tethering in the context of glycan biosynthesis is highlighted by a set of diseases known as congenital disorders of glycosylation, caused by defects in COG subunits [38,39]. It has also been shown that COG perturbation in CHO cells leads to a defect in O-linked glycosylation [40,41].

The recent development of robust genome editing tools enables us to interrogate host genes putatively associated with viral infections. These tools, such as the TAL effector nucleases (TALENs) [42,43] and CRISPR/Cas9 [44,45,46], can be programmed to target almost any DNA sequence and induce a double strand break (DSB). In the absence of an exogenous DNA template the DSB is primarily repaired by non-homologous end joining (NHEJ), often leading to frameshifting indels and gene knockout (KO). CRISPR/Cas9 relies on base pairing between the 20-bp seed sequence of the guide and target DNA for specificity, a unique characteristic that enabled the development of CRISPR libraries targeting all protein coding genes in humans, mice and the cow, facilitating the study of genes pivotal to host-pathogen interactions in a high throughput manner [47,48,49,50]. As an alternative to gene KO, Cas9 can also be repurposed to either upregulate or downregulate endogenous genes. To achieve this the two endonuclease domains of the Cas9 protein are deactivated to produce a catalytically “dead” Cas9 (dCas9), and gene transactivating or inactivating domains are fused to this to activate (CRISPRa) [51] or interfere (CRISPRi) [52] with gene transcription. For CRISPRi, a fusion complex consisting of dCas9, KRAB and the TRD domain of MeCP2 has proven to be efficient in gene silencing [52]. To ensure efficient down regulation of host gene expression, accurate Transcription Start Site (TSS) annotation is required as the guide RNA must be designed to bind immediately downstream of this sequence.

In a previous genome-wide CRISPR knockout study, we screened all protein coding genes in cattle to identify those that influence BoHV-1 replication [49]. Having compiled a list of host genes with potential involvement in the viral replicative cycle, here we further evaluated candidate genes most likely involved in viral entry, using a combination of single gene bi-allele KO and CRISPRi methods to test some of them. We validated the two receptors on the list, PVR and PVRL2, and their additive effect on mediating entry. In addition to genes coding for enzymes that directly catalyze biosynthesis of HS, we also studied the role the COG complex plays during virus entry.

## 2. Materials and Methods

All procedures or materials, if not listed here, such as nucleic acid extraction, RT-qPCR, and VP26-GFP growth monitoring with CLARIOStar, are available in the Supplementary Materials or described in detail previously [49].

### 2.1. Cells and Viruses

Wild type Madin-Darby bovine kidney cells (MDBK), Cas9+/+ MDBK cells or dCas9+/+ cells [49] were cultured in DMEM (D5796, Sigma, Tokyo, Japan) supplemented with 5% horse serum (26050088, Gibco), 1% Pen/Strep (15140122, Gibco), 1% L-glutamine (25030024, Gibco), 1% NEAA (11140035, Gibco, Paisley, Scotland), and 1% sodium pyruvate (11360039, Gibco). Cells were kept in an incubator set at 37 °C with 5% CO_2_. A GFP tagged BoHV-1 strain based on strain Jura [53] was used throughout the experiment. The GFP protein is fused to the VP26, a minor capsid protein of BoHV-1.

### 2.2. Plasmids

The dCas9 targeting vector was created by Gibson Assembly, incorporating the inducible dCas9-KRAB-MeCP2 fusion cassette from PB-TRE-dCas9-KRAB-MeCP2 (gift from Andrea Califano, Addgene # 122267), the EF1a promoter driven rtTA fragment from PB-CA-rtTA Adv (gift from Andras Nagy, Addgene # 20910), and an in-frame Hygromycin selection marker connected by 2A peptide sequence. These cassettes were flanked by ~1 kb left and right homology sequences amplified from the bovine rosa26 locus to enable HDR mediated knock-in. The PiggyBac vector used to deliver CRISPRi, PB-U6g5_PGK_Puro2aBFP, was constructed by cutting out the segment containing the hU6 sgRNA expression cassette and the Puro2aBFP selection marker from pKLV2-U6gRNA5(BbsI)-PGKpuro2ABFP-W (gift from Kosuke Yusa, Addgene # 67974) and cloning it into a PiggyBac backbone. The transposase expression vector pCMV-hypBase was shared by Dr. Kosuke Yusa while he was based at the Wellcome Sanger Institute. The H1 and mU6 donor plasmids were created by inserting PCR fragments with scaffold and H1/mU6 sequences into the Zero Blunt Topo vector.

### 2.3. dCas9 Knock-In to the Rosa26 Locus

The dCas9 fusion protein expression cell line dCas9+/+ was created by transfecting cells with 10 ug of the targeting vector and 1 ug TALEN 1.6 mRNA described previously [49]. After 3 days of culture at 33 °C, cells were selected with 500 ng/mL hygromycin. The process of isolating and expanding targeted single cell clones was completed as described previously [49].

### 2.4. Guide RNA Design

Please also refer to methods as described previously [49]. Briefly, guides used for generating the KO clones were selected from the btCRISPRko.v1 library [49]. The CRISPRi/3i guides were designed using a similar pipeline to that used for the btCRISPRko.v1, utilising the 400 bp genomic sequence immediately downstream of the TSS rather than protein coding sequence. The TSS information was extracted and combined from both the Ensembl (release-95) and NCBI (GCF_002263795.1) annotations of the Bos taurus assembly ARS-UCD1.2. For genes with two TSS annotations within 1 kb of each other, the downstream TSS was chosen. For genes with the two predicted TSS annotations greater that 1 kb apart, the NCBI annotation was used for the design. Please refer to Tables S1 and S4 of the Supplementary Materials for guide RNA sequences.

### 2.5. CRISPR3i Vector Cloning

To prepare the fragments, the dsODNs containing the guides were annealed by mixing two complementary oligonucleotides and cooling from 95 °C to 25 °C at 0.1 °C/s; the vector backbone and H1 mU6 donor plasmid were digested with BbsI and fragments of the predicted sizes were gel extracted. The six fragments (Figure 7b) were ligated using T4 ligase then transformed into Stabl3 competent cells. Colonies were pre-screened with bacterial PCR and Sanger sequencing verified before plasmid purification. For a more detailed protocol for the cloning, please refer to the Supplementary Materials.

### 2.6. Transfections

All transfections were done using a Neon electroporator with parameters as follows: voltage, 1200 v, duration: 30 ms, pulses: 2. To generate KO clones, 5 × 105 Cas9+/+ cells were loaded into a 100 uL Neon tip and electroporated with 1 ug of gene specific sgRNA transcribed in vitro as previously described [49]; for CRISPRi/3i targeting, PB vector carrying CRISPRi expression cassettes and pCMV-hypBase were mixed at 3 ug:1 ug ratio prior to electroporation.

### 2.7. Generation of KO Clones and Stable Cells with CRISPRi/3i KD

For KO clones, Cas9+/+ cells was transfected with 1 ug in vitro transcribed guide and after three days of culture, cells were plated at 50–100 cells/10 cm dish. A week after plating, clones were picked, expanded and genotyped by Sanger sequencing and TIDE analysis [54] (Table S3). For cells with CRISPRi/3i KD: the dCas9+/+ cells were transfected as described above, recovered with two days incubation and selected with Puromycin(1.8 ug/mL) for a week. Doxycycline was added to the culture media at 500 ng/mL for two days or throughout the plaque assay period to induce sgRNA expression and KD.

### 2.8. Viral Infections and Plaque Assays

For viral infections, viral stocks were defrosted in a 37 °C water bath and serial diluted in full growth media with 2% horse serum. 1 mL of diluted virus was added to each well of a 6-well plate and rocked gently to evenly distribute the virus. 1 h after the addition of virus, the viral supernatant was removed and 2 mL of fresh medium supplemented with 5% horse serum was added; this time point was regarded as zero hours post infection or 0 h.p.i. The MOI and time points for each individual experiment are specified in the legends or the accompanying texts.

For plaque assays, 2 mLs of medium with 2% horse serum and 0.5% Avicel was added at 0 h.p.i instead and the plates were incubated for four days prior to fixation and staining. The plates were scanned using an Epson document scanner and the sizes of plaques (total area) measured using ImageJ software.

To assess titer of virus grown in infected cells, supernatant and cell pellet samples were harvested from each well at specified time points. The supernatant was used to infect non-modified Cas9+/+ cells directly, whereas the pellets were resuspended in 500 uL PBS, frozen and thawed once and the spun supernatant used for plaquing.

For the attachment and entry assays, cell lines with PVR-/-;/PVRL2-/-, COG6-/-, COG7-/-, HS2ST1-/- genotypes (three distinct clones for each gene, Table S3 of the Supplementary Materials), Cas9+/+ (clone P) or WT cells were plated on 96-well plates with 25,000 per well. The day after plating, cells were challenged with the GFP tagged virus diluted in ice cold full media at MOI = 1. The plates were then incubated on ice for 1 h to synchronize attachment. After synchronization, cells on all plates were washed with ice cold PBS 3x and moved back to the 37 °C incubator in full growth media; this time point was regarded as 0 h.p.i. At 3 h.p.i., plates were washed with either PBS or an acid wash buffer (40 mM Na citrate, 135 mM NaCl, 10 mM KCl, pH 3.0) for one minute and then returned to the incubator in full growth media for continued infection. At 16 h.p.i., total virus samples were harvested and subsequently titrated on wild type MDBK cells by plaque assays.

### 2.9. Genome/Pfu and VP26/Pfu Ratio Experiment

To calculate the genome/pfu ratios of virus samples described in 2.10, the BoHV-1 genome copy number was obtained for each sample first. 5 uL of each virus was lysed by 5 uL QuickExtract DNA lysis buffer according to manufacturer instructions (EPICQE09050, Epicentre). The BoHV-1 DNA in each sample was then quantified by qPCR using UL23 primers listed in Table S2. The qPCR was performed in a Bio-rad CFX96 qPCR machine using LuminoCt SYBR Green qPCR ReadyMix (L6544, Merck). The genome copy number of each sample was calculated as 2^-(Ct – Av(P)) with Av(P) being the average Ct value of viruses produced by the Cas9+/+ cells. The genome/pfu ratio was then calculated for each sample, and the average ratio of viruses growth in the Cas9+/+ was chosen as the normalization factor and used to divide each genome/pfu ratio. These normalized genome/pfu ratios were used to plot Figure 5c.

To obtain the VP26/GFP ratios shown in Figure 5b, the VP26-GFP capsid proteins were quantified by densitometry, performed on the western blot images shown in Figure 5e using ImageJ. The ratio between VP26 and pfu was calculated for each sample and normalized by the average of ratios for the viruses grown in Cas9+/+ cells.

### 2.10. Lectin Staining and Microscopy of KO Cells

COG6-/-, COG7-/-, HST2ST1-/- and Cas9+/+ cells were plated on round coverslips on 24-well tissue culture plates at 35% confluency. One day later, cells were fixed with 4% Formaldehyde diluted in PBS for 10 min and then washed once with PBS. The cells were then blocked with 1× Carbon Free blocking buffer for one hour (SP-5040-125, Vectorlabs). This was followed by staining with Lectin GS-II From Griffonia simplicifolia, Alexa Fluor™ 647 Conjugate (L32451 from Thermo Fisher, Waltham, MA, USA), Lectin HPA From Helix pomatia (edible snail) Alexa Fluor™ 647 Conjugate (L32454 from Thermo Fisher), or Biotinylated Galanthus Nivalis Lectin (GNL, B-1245-2 from Vectorlabs) for one hour in room temperature. The GNL coverslip set was further stained by Streptavidin, Alexa Fluor™ 647 Conjugate (S32357 from Thermo Fisher). Afterwards, the stains were discarded, and cells were counter stained with Hoechst 33342 dye for 10 min in room temperature. All stains were diluted in the carbon free blocking buffer at 1:5000 and all washes were performed using PBS three times between steps. After the final wash with PBS, the coverslips were mounted on glass slides using Pro-long Gold anti-fade mounting solution. The slides were cured in room temperature overnight and visualized on a Leica DMRB upright fluorescence Microscope with a 40X objective lens.

### 2.11. Lectin Staining of Concentrated Viruses

To generate viruses for the lectin staining, the COG6-/- and Cas9+/+ cells were infected with the GFP tagged BoHV-1 virus at MOI = 0.1. 48 h later, 3 mL supernatant virus grown in the COG6-/- or control cells were spun at 45,000 rpm in a Beckman benchtop ultracentrifuge for one hour and resuspended in 30 uL PBS. From each sample, 5 uL of resuspended virus was then lysed with 2× Laemmli buffer with DTT at 95 °C for 5 min. The lysates were then resolved on a 4–20% gradient SDS PAGE gel at 120 V for 75 min and transferred onto a 0.2 um nitrocellulose membrane using the Bio-rad Trans-Blot Turbo Transfer System. The membrane was then blocked with the 1× Carbon Free blocking buffer (Vectorlabs) for an hour. Afterwards, the membrane was blotted against the VP26-GFP protein with an anti-GFP antibody (raised in mouse, 1:2000 dilution, JL-8, Takara Bio, Kusatsu, Shiga) and a lectin stain (1:5000 diluted GS-II or HPA stain as specified above in 2.9). The blot was further blotted with a Donkey anti-mouse IgG secondary antibody to label the JL-8 primary antibody (926-32212, Licor, Lincoln, NE, USA). All washes between steps were performed using PBST (PBS buffer plus 0.1% Tween-20) and after the final wash the blot was scanned with a Licor Odyssey Fc imaging system.

### 2.12. Statistical Analysis

All pairwise comparisons between the control cells (wild type or Cas9+/+ cells) and KO clones were done using *t*-tests with *p*-value cut-offs as follows: ns, *p* > 0.05; *, *p* ≤ 0.05; **, *p* ≤ 0.01; ***, *p* ≤ 0.001; ****, *p* ≤ 0.0001. For the time course experiment in Figure 4d, a two-way ANOVA followed by multiple pairwise comparisons were conducted instead.

## 3. Results

### 3.1. Genome-Wide CRISPRko Screen Identified Many Host Genes Potentially Important for BoHV-1 Entry into the Cell

Previously we conducted genome wide CRISPR Knockout screens during BoHV-1 replication in Madin-Darby bovine kidney (MDBK) cells [49]. In these screens, we challenged CRISPR library transduced cells with a GFP tagged BoHV-1 virus (GFP fused to minor capsid protein VP26) and then FACS sorted the cells into lowly and highly infected. Upon sequencing these cell fractions from the sort by next generation sequencing, we identified genes with enriched or depleted CRISPRs, to which pro- or anti-viral roles were assigned. Out of all the candidates, our CRISPRko screen identified at least 41 pro-viral host genes potentially related to virus entry.

**Figure 1:**
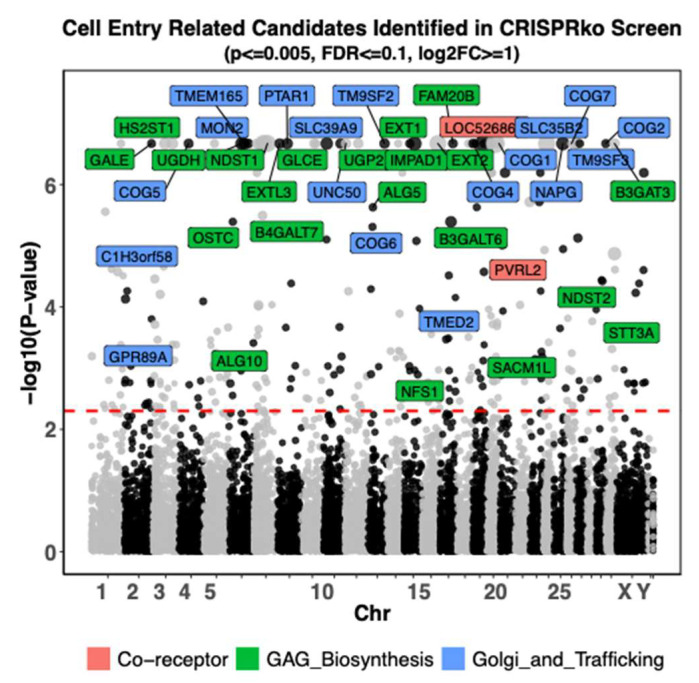
Genome-wide CRISPR knockout screen identifies pro-viral genes associated with viral entry into the cell. Guide RNA copy number changes obtained from the CRISPRko screen by comparing GFP Negative cells to GFP High cells [49] were plotted. Each dot represents one of the 21,216 protein coding genes targeted by the btCRISPRko.v1 library, with its genomic location plotted against the *x*-axis and −log10 (*p*-value) based on MAGeCK76 analysis against the *y*-axis; the sizes of dots represent -log2FoldChange values. Pro-viral candidate genes that are most likely to affect BoHV-1 cell entry with *p*-value ≤ 0.005, False Discovery Rate (FDR) ≤ 0.1, and log2 fold changes (log2FC) ≥ 1 as statistical cutoff are highlighted; they are color coded based on the pathways they might impact. The red line represents *p*-value threshold as 0.005 or −log10(*p*-value) = 2.3. Chr: short for chromosome, due to space constraint, some chromosome numbers were omitted.

The proteins encoded by these candidate genes fall into three major categories: cell surface receptors, enzymes involved in GAG biosynthesis and proteins involved in trafficking within the Golgi apparatus (Figure 1, Figure S1 of the Supplementary Materials). The two receptor encoding genes we recovered are LOC526865 (PVR or CD155) and PVRL2 with the latter being suspected as a receptor but never experimentally confirmed [24]. The screen also captured the prime importance of GAG for BoHV-1 entry into the cell, with 22 of the candidates associated with HS biosynthesis. In addition to enzymes known to catalyse HS synthesis [55], other candidates have been found to affect HS levels and impact entry of viruses such as Chikungunya Virus [56], Lassa virus [57], Rift Valley Fever Virus [58], and Vaccinia Virus [59]. Some of these proteins are important for N-glycosylation, including Glycosyltransferases ALG5 and ALG10, and subunits of the Oligosaccharyltransferase complex OSTC and STT3A. Other candidates that may transport or catalyse formation of crucial precursor molecules for HS were also selected, namely GALE, NFS1, PTAR1, SLC35B2, SLC39A9 [57,58], UGDH and UGP2. In the third category, six genes encoding subunits of the COG complex [37] (COG1, COG2, COG4, COG5, COG6, and COG7) were identified. Other genes encoding resident proteins of the Golgi with demonstrated or suspected indirect roles in protein glycosylation [56,57,58,59] (C1H3orf58, IMPAD1, MON2 [60], NAPG, SACM1L, TM9SF2, TM9SF3, TMED2, TMEM165 and UNC50) were also enriched.

### 3.2. Knockout of PVR and PVRL2 Impaired BoHV-1 Attachment and Entry into the Host Cell

**Figure 2:**
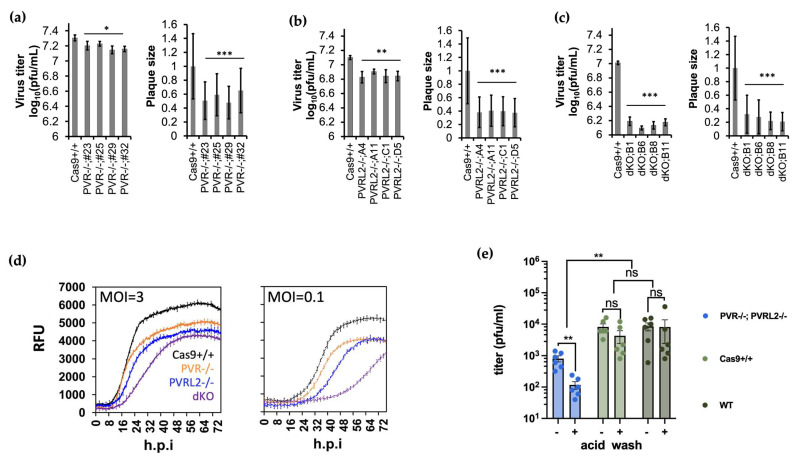
Loss of cell surface receptors impair BoHV-1 replication in MDBK cells. (**a**). Plaque assay results in four clones with PVR (a.k.a LOC526865) KO compared to Cas9+/+ control cells. (**b**). Plaque assay results in four clones with PVRL2-/- compared to Cas9+/+ controls cells. (**c**). Plaque assay results in four clones with PVRL2 and PVR double KO (dKO) compared to Cas9+/+ controls cells. All plaque assays shown in (**a**–**c**) were done with at least two biological repeats (n ≥ 2), while plaque sizes were averages measured from at least 40 plaques and then normalized to those from Cas9+/+ cells. (**d**). BoHV-1 VP26-GFP protein growth curves in Cas9+/+, PVR-/-, PVRL2-/-, and PVR/PVRL2 dKO cells infected at MOI = 3 or 0.1 measured as fluorescence intensity (n = 4) throughout a 72-h infection. (**e**). Titers of total viruses harvested from non-KO cells (Cas9+/+ or WT cells) and PVR-/-; PVRL2-/- dKO cells at 16 h.p.i.(n = 2 with three clones each). More details described in 2.8. Briefly, ice cold virus inoculum was added at MOI = 1 to each cell line and the plates were incubated on ice. 1 h later (a.k.a. 0.h.p.i.), the inoculum was taken off and the cells were washed with ice cold PBS thrice before being returned to the 37 °C incubator. 3 h later, cells were washed again with an acid buffer (pH = 3) or PBS for 1 min and the plates were returned to the incubator till harvest. All analyses based on pairwise *t*-tests with ns: not significant with *p* > 0.05; *: *p* ≤ 0.05; **: *p* ≤ 0.01; ***: *p* ≤ 0.001; error bars represent +/- 1 SD.

We initiated our validation efforts by testing receptor candidates for BoHV-1, PVR and PVRL2. PVR has been indicated as an entry receptor for BoHV-1 while PVRL2 has only been hypothesized to be so, and its candidacy has never been properly tested [14,16]. To achieve this, we first generated biallelic knockout (denoted as -/- hereafter) cell clones lacking PVR by transfecting in vitro transcribed sgRNAs targeting this gene into a MDBK cell line expressing Cas9 (Cas9+/+) [49]. When we directly titrated BoHV-1 on these PVR KO cells, we detected a 1.7-fold drop in apparent virus titre and a 2.1-fold decrease in plaque size compared to the parental Cas9+/+ cells (Figure 2a). When we conducted the same experiment targeting PVRL2 instead, the impact on the virus was more pronounced. By plaquing directly on these KO cells, we detected an up to 1.9-fold decrease in virus titre and 2.7-fold reduction in plaque size compared to the control cells (Figure 2b). Although the impact on virus replication caused by loss of either receptor alone was statistically significant, the effect size was rather modest. This is likely due to the ability of alpha herpesviruses to bind to multiple cell surface molecules to mediate entry. Therefore, we generated double KO (dKO) clones lacking expression of both PVRL2 and PVR and examined virus replication. Based on plaque assays, the impact of the dKO was more prominent than the sum of the individual gene KOs, with virus titre and plaque size reduced by up to 8.2-fold and 4.8-fold respectively compared to the parental Cas9+/+ controls (Figure 2c).

We then studied the rates of viral protein synthesis by infecting them or control cells with a GFP-tagged BoHV-1 [53] and sequentially recording GFP intensity for 72-h. We detected both a delay and a reduced magnitude of VP26-GFP expression in all KO cells (PVR-/-, PVRL2-/- and particularly the dKO cells) compared to the parental Cas9+/+, when cells were infected at either MOI = 3 or MOI = 0.1 (Figure 2d). Taken together, these results confirm that both PVR and PVRL2 can serve as receptors for BoHV-1 and that they work cooperatively for the virus. Our data also consistently suggest that PVRL2 mediates more efficient entry than PVR in MDBK cells. Guides targeting PVRL2 were more enriched in the CRISPRko screen [49] and its loss led to a more severe impact on viral replication manifested by greater loss of viral titre and spread (Figure 2a,b), as well as a larger reduction and delay in viral protein synthesis (Figure 2d).

To further understand the mechanism behind reduced plaquing efficiency and VP26-GFP growth in these knockout cells, we conducted viral attachment and entry assays on them. To achieve this, we first infected the PVR-/-;PVRL2-/- dKO cells and control cells with ice cold virus at MOI =1. We then synchronized viral attachment to cells by incubating the infections on ice (Figure 2e). One hour later (0 h.p.i.), we took the inoculum off from each well and washed the cell monolayer with ice cold PBS three times, before returning it to the 37 °C incubator in full growth media. This should remove viruses that failed attachment to cells. Three hours later, we further washed the cells with an acid buffer (pH = 3.0) for 1 min, to remove viruses that had not penetrated the cellular membrane, or with PBS as controls. After a single cycle of infection, we harvested total virus from each well at 16 h.p.i. and titrated it on WT MDBK cells.

We found that less virus was produced by the dKOs in both conditions tested (-/+ acid wash, Figure 2e), compared to the control cells (Cas9+/+ and WT cells). Without acid washes, there was a 10-fold reduction in virus titers produced by the dKO cells relative to those by the Cas9+/+ cells, suggesting that fewer viruses attached themselves to the dKO cells than to the non-KO cells during the on-ice incubation. Furthermore, the acid wash at 3h.p.i. had no impact on virus production by the Cas9+/+ or WT cells, indicating that under normal conditions (cells being intact without KO), all viruses had successfully internalized after 3 h. On the contrary, there was a further reduction (7-fold) in virus production by the dKO cells that undergone acid wash relative to those that did not, suggesting a significant delay in entry of the viruses into the dKO cells compared to the control cells. To summarize, these reductions indicate that the dKO negatively impacted the virus on two fronts, on both the attachment and the entry into these host cells missing PVR and PVRL2, further suggesting that PVRL2 is a receptor for BoHV-1.

### 3.3. Heparan Sulfate but Not Chondroitin Sulfate Promotes Efficient BoHV-1 Entry

**Figure 3:**
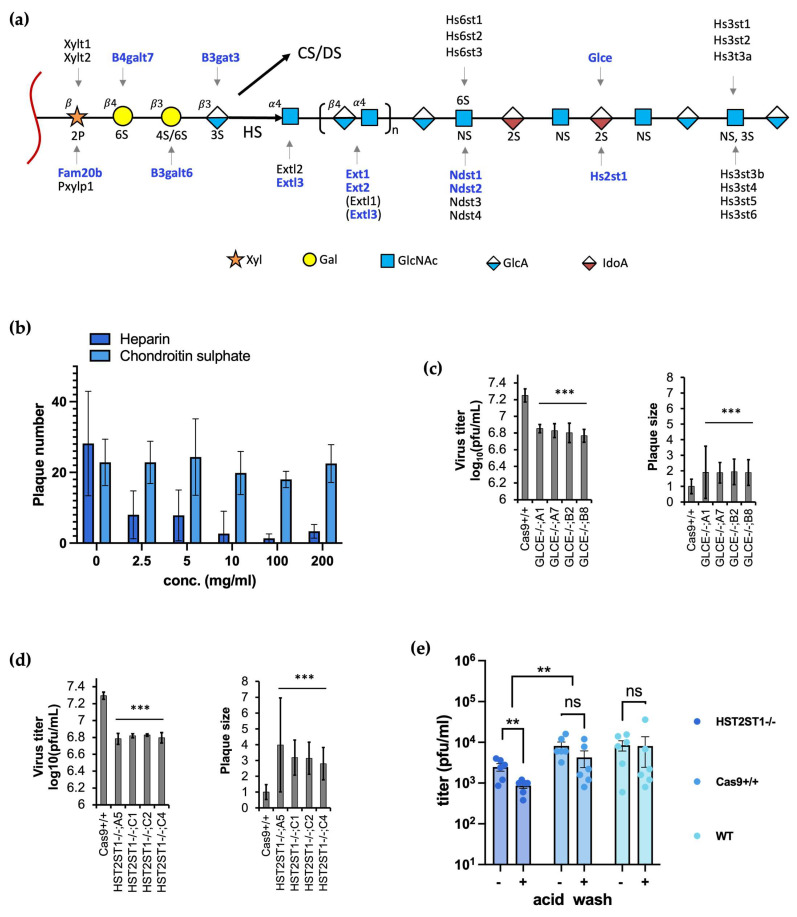
Many candidates catalyze cell surface Heparan Sulfate proteoglycan synthesis. (**a**). Genes known to be involved in each step Heparan Sulfate (HS) biosynthesis and chain extension from the core protein (red squiggle); those highlighted in blue were identified by the screen. This diagram is modeled after Figure 2 in Chen et al. [55] with the pathway to chondroitin sulfate (CS)/dermatan sulfate (DS) synthesis simplified. Abbreviations for units within the HS chain: Xyl, xylose residue; Gal, galactose residue; GlcNAc, 2-deoxy-2-acetamido-α-D-glucopyranosyl; GlcA, β-D-glucuronic acid; IdoA, α-L-iduronic acid. (**b**). Cells were treated with specified concentrations of Heparin or Chondroiten Sulfate prior to infection by BoHV-1. Total viral samples were harvested and titrated on wt MDBKs cells (n = 3 for HS blocking assay and n = 2 for CS blocking). (**c**). Plaque assay results from four GLCE-/- clones, A1, A7, B2 and B8 compared to Cas9+/+ control cells (n ≥ 6). Plaque size measurements were taken from at least 50 plaques for each clone and then normalized to those from Cas9+/+ cells. (**d**). Plaque assay results from four HST2ST1-/- clones, A5, C1, C2 and C4 compared to Cas9+/+ control cells (n ≥ 5). Plaque size measurements were taken from at least 89 plaques for each clone and normalized to Cas9+/+ cells. (**e**). Titers of total viruses grown in non-KO cells and HST2ST1-/- KO cells (MOI = 1) harvested at 16 h.p.i. The experiment was conducted the same as described in Figure 2e and Section 2.8. All analyses based on pairwise *t*-tests with ns: not significant with *p* > 0.05; **: *p* ≤ 0.01; ***: *p* ≤ 0.001; error bars represent +/− 1 SD.

Like many other viruses, BoHV-1 initiates entry by first interacting with cell surface HS. Out of the 41 viral entry associated candidates from the CRISPRko screen, at least 11 genes encode for enzymes that directly catalyse GAG linker assembly or HS chain initiation, elongation, and modification (Figure 3a, genes identified by the screen are highlighted in blue). Four of these genes are crucial for steps of common chain linker assembly for both HS and CS/DS, including glycosaminoglycan xylosylkinase (FAM20B), galactosyl-transferases (B4GALT7 and B3GALT6), and glucanosyltransferase (B3GAT3) (Figure 3a). After linker assembly, the biosynthesis pathways begin to diverge, and different enzymes are required for HS and CS/DS chain initiation and elongation. The screen also identified EXTL3, one of the two glycosyltransferases known to initiate HS chain formation and which has been experimentally shown to be more efficient for this purpose than the other enzyme EXTL2 (Figure 3a) [35,61]. HS chain elongation is thought to be achieved by exostoses genes EXT1 and EXT2, both identified in our screen, with KO of either of these genes completely abolishing HS synthesis [55].

As the HS chain polymerizes, the nascent heparosan chain undergoes a series of sulfation and epimerization carried out by four classes of sulfotransferases and an epimerase [25]. Our screen identified NDST1 and NDST2 (Figure 3a), two of the four genes encoding homologous bifunctional N-deacetylase–N- sulfotransferases de-N-acetylates. While experiments in CHO cells showed that KO of either gene alone did not lead to substantial reduction of disaccharide sulfation [55], when both genes were deleted simultaneously disaccharide sulfation was eliminated. In addition, the one epimerase that converts GlcA to IdoA (Figure 3a) encoded by GLCE was also identified in the screen, along with HST2ST1, a 2-O-sulfotransferase that is predicted to interact with the protein product of GLCE [62].

To experimentally confirm the positive role of HS in cell entry, we first conducted Heparin blocking assays by treating cells with different concentrations of Heparin during a BoHV-1 infection then titrating viruses produced from these cells on wild type MDBK cells; there was a steady decrease in virus titre as Heparin concentration increased (Figure 3b). We then produced KO clones lacking either GLCE or HST2ST1 and assessed viral replication in them. The loss of either gene alone led to average 2.7-fold (GLCE) and 3.1-fold (HST2ST1) reduction in apparent virus titre when they were used for plaque assays (Figure 3c,d). Surprisingly, although fewer plaques were produced, their size was increased in both cases, up 1.9-fold in GLCE-/- cells and 3.3-fold in HST2ST1-/- cells compared to Cas9+/+ controls (Figure 3c,d). Taken together, these results demonstrate the important roles various genes, such as GLCE and HST2ST1 play during BoHV-1 replication by catalysing HS synthesis.

To confirm and further understand the role HST2ST1 plays during BoHV-1 replication, we conducted another attachment and entry experiment, utilizing the HST2ST1-/- KO cells. With a similar virus infection setup as described for the PVR-/-;PVRL2-/- cells (Figure 2e), we synchronized virus attachment to the HST2ST1 KO and control cells on ice for an hour, treated them with the acid wash at 3h.p.i., then harvested the total viruses produced by these cells at 16h.p.i. for titration. We observed similar trends (Figure 3e) as seen in the PVR-/-;PVRL2-/- experiment (Figure 2e), in both conditions (-/+ acid wash) the titers of viruses produced by the HST2ST1-/- cells were lower compared to the control cells. However, the effect sizes were smaller; there was a 3.3-fold drop in virus titer without acid wash (10-fold for PVR-/-;PVRL2-/- dKO) and another 2.6-fold reduction was recorded with the acid wash (7-fold for the dKO). These data indicate that like the PVR/PVRL2 dKO, the loss of HST2ST1 also affected both the attachment and entry of BoHV-1 into these deficient cells. However, the smaller effects might suggest that HST2ST1 is less important for the virus than the receptors.

As a further note, despite identifying many genes crucial for HS production, no enzymes specific for CS chain initiation, extension or modification showed up in the screen [55]. This indicates that even though CS has been shown to play an auxiliary role in cell surface binding by HSV-1 [32,63], it is not required for BoHV-1 to mediate entry into MDBK cells, a conclusion supported by our chondroitin sulfate blocking assay (Figure 3b).

### 3.4. COG KO Impaired Multi-Cycle but Not Single Cycle BoHV-1 Virus Replication

**Figure 4:**
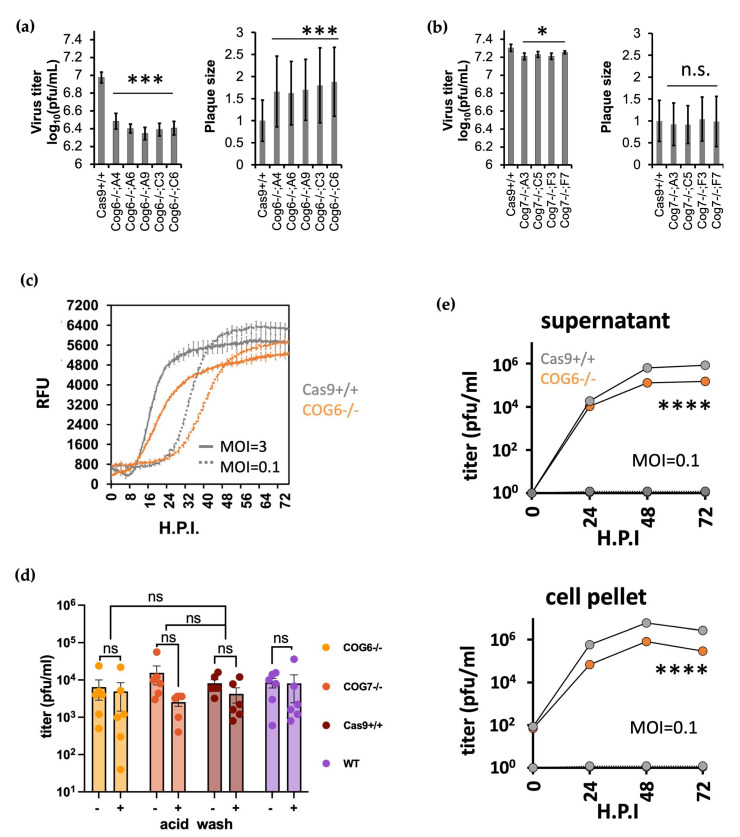
The loss of COG6 and COG7 affects BoHV-1 replication in MDBK cells. (**a**). Plaque assay results from five COG6-/- KO clones compared to Cas9+/+ control cells with measured virus titers (left) and plaque sizes (right) shown. (**b**). Plaque assay results from four COG7-/- KO clones compared to Cas9+/+ control cells with measured virus titer(left) and plaque sizes shown (right). Statistics for A and B as follows: all plaque assays were conducted at least three times for each clone (n ≥ 3) with plaque sizes averaged from ≥ 65 plaques for each KO clone and then normalized to that from Cas9+/+ cells. ***: *p* < 0.005; *: *p* < 0.05; n.s.: not significant based on one-way ANOVA followed by multiple comparisons between Cas9+/+ and Cog7-/- clones, error bars represent +/− 1 SD. (**c**). VP26-GFP fluorescence growth curve in Cas9+/+ and COG KO cells infected with GFP tagged BoHV-1 at MOI = 3 or 0.1. (**d**). Plaque assay results by titrating virus harvested from COG6-/- KO or Cas9+/+ cells infected with BoHV-1 at MOI = 0.1 on wt MDBK cells, supernatant and cell pellet samples were collected at 0,24,48,72 h.p.i. Two-way ANOVA followed by Šídák’s multiple comparisons test was performed to compare the two cell lines (n = 3, ****: *p* < 0.0001). The error bars (+/− 1 SD) are too small to be displayed. (**e**). Titers of total viruses grown in non-KO cells and COG6 or COG7 KO cells (MOI = 1) harvested at 16 h.p.i. The experiment was conducted the same as described in Figure 2e and Section 2.8. All analyses except the time course experiment (**d**) based on pairwise *t*-tests with ns: not significant with *p* > 0.05; *: *p* ≤ 0.05; ***: *p* ≤ 0.001; ****: *p* ≤ 0.0001; error bars represent +/- 1 SD.

To evaluate the importance of the COG complex to BoHV-1 replication, we first generated single gene KO clones of either COG6 or COG7. Plaque assays revealed that KO of either gene reduced virus titer in multiple clones, with up to 4.3-fold and 1.5-fold decrease respectively (Figure 4a,b). Interestingly, the sizes of plaques grown in COG6 -/- cells increased to at least 1.6-fold (Figure 4a), whereas no significant augmentation was observed in COG7 -/- clones (Figure 4b). A similar reduction in virus titre was also observed in the COG6 -/- cells when we conducted plaque assays directly on these cells with serial diluted Alcelaphine Virus 1, a gamma herpes virus endemic to wildebeest that causes malignant catarrhal fever when transmitted to cattle (Figure S2 of the Supplementary Materials) [64]. The negative impact on BoHV-1 replication associated with the loss of COG6 expression was further evidenced by delayed production of the VP26-GFP protein after high or low MOI infection of COG6-/- KO cells with BoHV-1 VP26-GFP virus compared to the Cas9+/+ control cells (Figure 4c). To dissect this phenomenon, we harvested supernatant and cell pellets from the virus infected Cas9+/+ and COG6 -/- cells and titrated these on wild type MDBK cells. There were significantly fewer infectious viral particles in both the supernatant and cell pellets collected from infected KO cells compared to parental Cas9+/+ cells, at the end of a 72 h infection (Figure 4d). These results confirm the positive role the COG complex plays during BoHV-1 replication.

We then conducted a series of attachment and entry assays to see if the COG6 and COG7 KO took effect on BoHV-1 replication during these processes, as with the PVR-/-; PVRL2-/- dKO and HST2ST1-/- KO cells (Figure 2e and Figure 3e). After infecting the COG KO cells at a MOI =1, synchronizing attachment on ice for 1 h and washing them with the acid buffer at 3 h.p.i., we collected total virus from these KO cells at 16 h.p.i. for titration. To our surprise, we didn’t detect any significant difference among viruses produced by the COG6-/-, COG7-/- and the control cells, regardless of the acid wash (Figure 4e). These negative results, obtained after single cycle infections from COG6 and COG7 KOs, are contrary to the significant reductions of virus titers collected from the same cells after multi-cycle infections (Figure 4a,b,d). Taken together, these results indicate that during the multi-cycle infections, the first cycle of replication was not affected in the COG KOs, and the negative impact of COG KO only manifested during the subsequent cycles of replication.

### 3.5. COG6-/- Cells Produced Less Infectious Viruses Compared to Those by Wildtype Cells

Since the COG6-/- KO impacted the virus production during multi-cycle but no single cycle replications, we hypothesized that the KO only took effect after the initial cycle of replication, when new virions started being produced. We also conjectured that due to the KO, the new viruses produced by these cells were less infectious than those produced by the intact cells, leading to the compound effect seen with multi-cycle infections (Figure 4a,d). To test this, we first examined the particle/pfu ratios of viruses produced by the COG6-/- cells, as a proxy measurement of their infectivity. We collected supernatant viruses from COG6-/- cells and the Cas9+/+ cells at 48 h.p.i., after infection with MOI of 0.1. We then titrated these viruses on WT MDBK cells using two sets of plaque assays, one with 3 × PBS washes an hour post inoculation, while for the other set the inoculum remained throughout the plaquing process.

**Figure 5:**
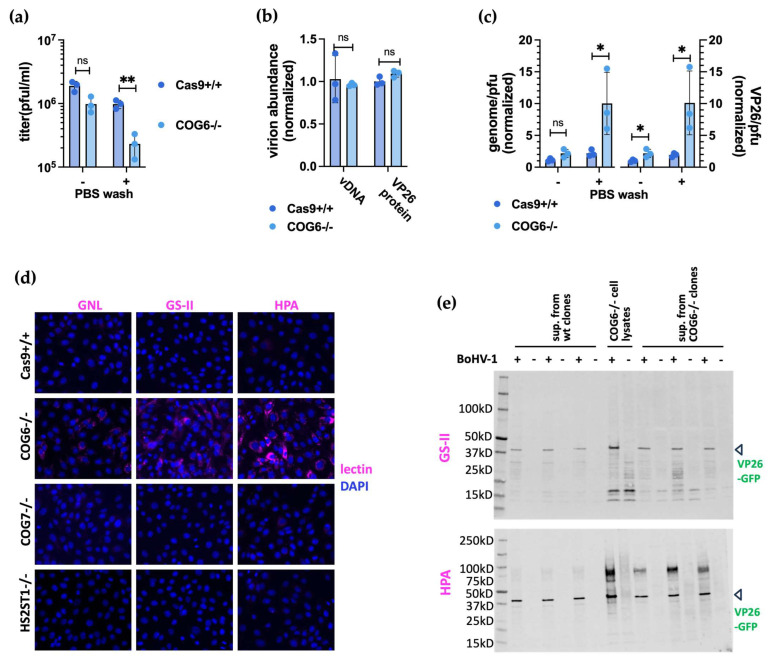
The loss of COG6 lead to the production of less infectious viruses possibly due to reduced glycosylation of viral protein(s). (**a**). Titers of supernatant viruses harvested (at 48 h.p.i. with MOI = 0.1) from COG6-/- or Cas9+/+ cells plaqued on wild type MDBK cells. For one set of the plaque assays, the inoculums were taken off one hour after addition of the viruses and the MDBK monolayers were washed three times with PBS. For the other set, the inoculums remained. (**b**). Quantification of viral particles in the samples based on the viral genome copy number (vDNA, measured by qPCR) and the VP26 protein abundance (measured by densitometry of western blot results shown in (**e**)). (**c**). Normalized genome/pfu ratios and VP26 protein/pfu ratios of these viruses. (**d**). Lectin staining results of the COG6-/-, COG7-/- and HST2ST1-/- clones and Cas9+/+ cells using GNL, GS-II and HPA dyes. The staining was visualized by fluorescent microscopy with positive lectin stain shown in pink while DAPI counterstain of the nuclei in blue. Representative results from three independent clones shown. (**e**). Lectin staining (GS-II and HPA) coupled with western blotting (against VP26-GFP) of concentrated viruses from (**a**). Concentrated supernatants from MOCK infected clones and COG6-/- cell lysates served as controls. Results shown are from three independent clones for COG6-/- and Cas9+/+ (wt). All analyses are based on pairwise *t*-tests with ns: not significant with *p* > 0.05; *: *p* ≤ 0.05; **: *p* ≤ 0.01; error bars represent +/− 1 SD.

Interestingly, while we failed to detect any significant different between viruses collected from the KO and Cas9+/+ control cells when the input virus remained. However, when the inoculum was removed, we recorded a significant 4.2-fold drop in titer of viruses collected from the KO cells (Figure 5a). By qPCR quantification of the viral genome (Figure 5b) and later by western blot of the VP26-GFP, the amounts of virions produced from the COG6-/- cells and the controls were the same (Figure 5b,e). Consequently, the calculated genome/pfu and VP26/pfu ratios were ~5-fold higher for viruses harvested from the KO cells than that from the control cells (Figure 5c). This translates to viruses harvested from the KO cells being only 20% infectious as those harvested from the Cas9+/+ cells, only under the PBS wash condition. These data further indicate that infection by viruses harvested from the KO cells is a delayed process.

### 3.6. COG6-/- Lead to Reduced Glycosylation Patterning of Host and Viral Proteins

The COG complex has been shown to regulate the Golgi glycosylation machinery. COG deficiency leads to glycosylation defects in cells, as previously indicated by elevated cell surface lectin staining [65]. To understand the reduced infectivity of viruses harvested from COG6 KO cells, we wondered if it was caused by insufficient glycosylation of viral proteins. To determine this, we first examined glycosylation of host cell surface proteins in the KO cells. We thus stained non-permeabilized COG6 KO cells with three lectins: lectin GNL that preferentially binds to structures containing terminal mannose, lectin GS-II from Griffonia simplicifolia that selectively binds to terminal non-reducing a- or ß-linked N-acetyl-D-glucosamine, and lectin Helix pomatia agglutinin (HPA) that is specific for α-N-acetyl galactosamine residues. All three lectins gave substantially more staining on the surface of COG6-/- cells compared to the Cas9+/+ control cells (Figure 5d). Interestingly, no such elevated staining was observed for the COG7 and HST2ST1 KO cells. These results serve evidence that N-glycosylation in these COG6-/- cells is impaired, due to COG6 disruption.

Since viral proteins use the same host cell post translational machinery during replication, we became curious whether the glycosylation of viral proteins was also affected by the COG6 KO. To find out, we first concentrated the supernatant viruses harvested from the KO and Cas9+/+ control cells by ultracentrifugation, resolved them on a denaturing SDS PAGE gel, and transferred the viral proteins onto a nitrocellulose membrane. We then blotted the membrane with the same lectin stains used earlier (Figure 5d), while utilizing the VP26-GFP as loading controls as detected by an anti-GFP antibody in western blotting. Out of the two stains tested, the lectin stain HPA labeled protein(s) of viruses harvested from the COG6-/- cells at much higher efficiency than those from the control cells. Prominent smearing bands are detected around 100 kD for the viruses produced by the COG6-/- cells (Figure 5e). These results demonstrate that the loss of COG6 disrupted glycosylation of both the host and viral proteins.

### 3.7. CRISPRi Efficiently Knocked down Host Gene Transcription

**Figure 6:**
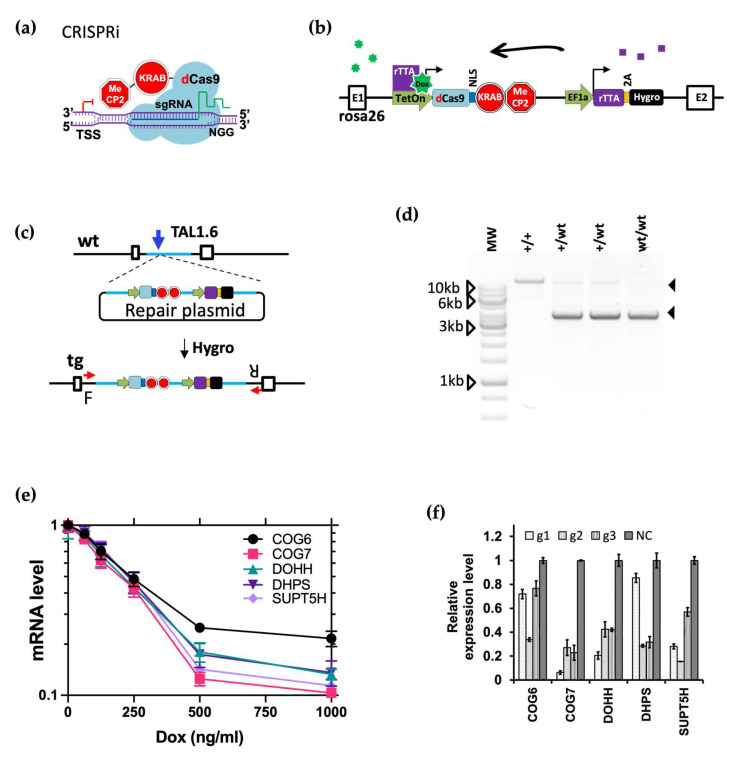
CRISPRi mediates efficient KD of gene expression in MDBK cells. (**a**). Schematic representation of the CRISPRi system applied in this study, based on the module developed by Andrea Califano et al. (See Materials and Methods). (**b**). Doxycycline inducible expression of the dCas9-KRAB-MeCP2 module from a stably integrated expression cassette at the cow rosa26 locus. Green heptagram represents doxycycline, purple squares are rTTA expressed from the downstream expression cassette, used to enhance doxycycline-driven induction. (**c**). Targeting strategy to knock-in the dCas9-KRAB-MeCP2 cassette into rosa26 using TAL1.6 stimulated HDR) [49] and Hygromycin selection, prior to dilutional cloning and genotyping by PCR using primer set F + R (red arrows) binding outside the homology arms. wt: wild type; tg: targeted. (**d**). Representative genotyping results by PCR from a homozygous targeted clone (+/+), and two heterozygotes (+/wt), black arrows point to amplicons from the targeted allele (top, 12 kb PCR product) and wt allele (bottom, 4 kb PCR product). (**e**). Knockdown efficiency of Doxycycline induced CRISPRi under six Doxycycline concentrations across five genes represented as mRNA level measured by reverse transcription and qPCR at 48 h post induction. (**f**). Relative expression levels of five genes in dCas9+/+ MDBKs each targeted by three CRISPRi guides (g1, g2, g3) compared to cells transfected with non-targeting CRISPRs (NC). These results are based on RT-qPCR results from a single experiment with three technical qPCR repeats, error bars represent +/- 1 standard error of the mean.

To facilitate candidate gene validation using an orthogonal method to gene KO, we decided to utilize the dCas9-KRAB-MeCP2 CRISPRi system that has been shown to knockdown gene transcription efficiently [52]. To achieve this, we first produced an alternative MDBK cell line with inducible expression of the dCas9 fusion protein (Figure 6a) by modifying the rosa26 locus with the fusion protein expressed from a doxycycline inducible TetOn promoter (Figure 6b). Using the same targeting and genotyping strategies employed to make the Cas9 expressing cell lines for the screen (Figure 6c) [49], we created a homozygous dCas9+/+ cell line for CRISPRi which was confirmed by genotyping PCR (Figure 6d).

Using these cells, we initially targeted five candidate genes, COG6, COG7, DOHH, DHPS, and SUPT5H, by introducing single guides designed to bind within a 400 bp region immediately downstream of the TSS of each gene and measuring mRNA transcript levels by reverse transcription and qPCR. Three guides per gene were cloned individually into the PiggyBac based doxycycline-inducible sgRNA expression vector PB-U6g5_PGK_Puro2aBFP (Materials and Methods). This vector was then co-transfected into dCas9 +/+ cells with the transposase plasmid pCMV-hypBase [61], allowing efficient stable transgene integration by transposition. Following puromycin selection for stable integrants, cells were cultured with doxycycline for 48 h prior to mRNA isolation, with 500 ng/mL as determined by a series of dosage experiments across five genes (Figure 6e and Figure S3 of the Supplementary Materials). Compared to control cells transfected with a non-targeting guide, candidate gene expression levels fell to between 6–85% of the basal levels in cells containing one of the three targeting guides (Figure 6f). For each gene at least one of the guides resulted in transcript suppression to below 40% of basal level. These results demonstrate that this CRISPRi system is very effective in suppressing gene expression in most cases.

### 3.8. CRISPR3i Enabled Rapid Validation of Candidate Genes

**Figure 7:**
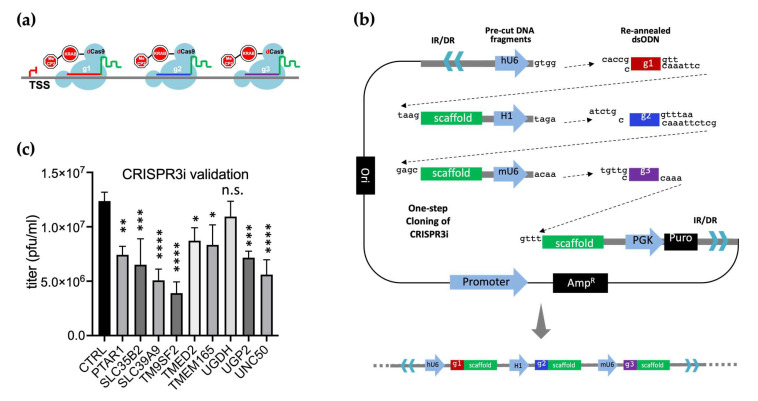
Multiplex CRISPRi against pro-viral candidate genes results in reduced viral titers. (**a**). CRISPR3i against a host gene in action by binding immediately downstream of TSS in tandem for synergistic gene repression. (**b**). The one-step cloning strategy to synthesize PiggyBac transposon vectors carrying three sgRNA expression cassettes in tandem to implement CRISPR3i by PB transposition. (**c**). Plaque formation assay results in cells with CRISPR3i expression intended to interfere with transcription of pro-viral genes involved in HS biosynthesis and other functions, as identified by the CRISPRko screen; CTRL represents virus titer from cells expressing three non-targeting sgRNAs (n >= 3). Results were ordered by *p*-value. ****: *p* < 0.0001; ***: *p* < 0.005; **: *p* < 0.01; *: *p* < 0.05; n.s. not significant with *p* > 0.05 based on one-way ANOVA followed by multiple comparisons between CTRL and CRISPR3i cells, error bars represent +/− 1 SD.

We reasoned that simultaneous expression of three guides tiled along the 400 bp downstream of a TSS (Figure 7a) might have a synergistic impact on candidate gene transcript suppression. To achieve reliable, coordinated expression of all guides in a transfected cell pool, they must all be delivered using a single construct. We therefore devised a one-step cloning protocol (the Supplementary Materials) that quickly assembles three sgRNA expression cassettes into the same PiggyBac based sgRNA expression vector used previously (Figure 7b). For the ligation, the linearized PiggyBac vector was mixed with three CRISPR containing short dsDNA fragments assembled from annealed oligonucleotides and two pre-cut fragments supplying sgRNA scaffolds and small RNA promoters hH1 and mU6 [62,63]. Unique overhangs at either end of fragments ensured orientation specific assembly. The resulting CRISPR3i vectors harbour an array of three sequential sgRNA expression cassettes, each with a unique promoter.

We used CRISPR3i to assess nine candidate genes, including multiple ones associated with GAG biosynthesis. These genes might transport or catalyse the formation of crucial precursor molecules for HS (PTAR1, SLC35B2, SLC39A9, UGDH and UGP2) [57,58] or encode resident proteins of the Golgi with potential and indirect roles in protein glycosylation (TM9SF2, TMED2, TMEM165 and UNC50) [56,57,58,59,60]. We constructed 10 CRISPR3i PiggyBac vectors, one targeting each candidate gene plus a negative control vector. CRISPR3i constructs were each co-transfected with pCMV-hypBase into dCas9+/+ MDBK. Cell pools were selected with puromycin then induced with doxycycline 48 h prior to plaquing the BoHV-1 virus directly on these cells. Doxycycline was added to the overlay throughout the incubation period to maintain consistent host gene suppression. Analysis revealed that the CRISPR3i approach resulted in significant reduction of virus plaquing efficiency in eight out of the nine candidates (Figure 7c). The protein products of these genes could directly or indirectly affect HS biosynthesis, via COG related protein trafficking or other routes (Figure 1). These results confirm the pro-viral roles of most of the genes tested using CRISPRi modulated target gene KD.

## 4. Discussion

In this study, we experimentally validated multiple candidate genes related to viral attachment and entry, as identified previously by our genome wide CRISPR KO screen [49]. For the first time, we provide evidence that PVRL2 is indeed a receptor for BoHV-1 entry into bovine cells. We also found that, in the MDBK cell line at least, it likely mediates more efficient infectious virus production than the previously identified PVR, as PVRL2 KO had a bigger impact on virus replication than did PVR KO (Figure 2). While double KO of both PVR and PVRL2 further reduced viral replication, it failed to confer MDBK cells with complete resistance to infection. Previous studies demonstrated that by editing a single cell surface receptor, pigs could be engineered to become resistant to porcine reproductive and respiratory syndrome virus [66,67] and transmissible gastroenteritis virus [68] infection, but our data show that this is not an appropriate strategy to create animals that are fully resistant to BoHV-1. This is likely due to the presence of other surface receptors such as nectin-1 for BoHV-1 and/or entry via receptor-independent mechanisms.

Previously our CRISPRko screen also identified key enzymes involved in multiple steps of HS synthesis (Figure 3a), and here we confirmed the significance of cell surface HS in mediating BoHV-1 entry. Interestingly, MDBK clones missing HS2ST1 or GLCE produced fewer plaques as expected, but surprisingly those that were formed were bigger compared to the parental controls. One possible explanation is that while lack of HS results in fewer initial infections and thereby lower plaquing efficiency, once an infection becomes established newly synthesized viral particles distribute more widely in the cell culture due to the reduced electrostatic attraction to the plasma membrane of adjacent cells. Indeed, it has been observed that multiple herpesviruses including HSV-1, HSV-2 and BoHV-1 upregulate heparinase transcription and expression, thereby promoting viral release [69,70]. The 3-O-sulfated disaccharides in HS are created by seven (Figure 3a) glucosaminyl 3-O-sulfotransferases in mammals, six of which are known to generate a binding site for gD of HSV-1 in humans [30]. This high functional redundancy is the likely reason why none of these genes was identified by the KO screens.

Another prominent cluster of candidates revealed by the screen encode six subunits of the COG complex. Reduced viral titer and delayed viral replication in COG6 -/- MDBK cells verified its pro-viral roles in BoHV-1 replication (Figure 4a–e). Interestingly, as with HST1ST2 and GLCE KO cells, plaques associated with COG6 KO cells were bigger than in the parental controls; the reduced cell entry but possible faster release of BoHV-1 in these KO cells strongly indicates disrupted HSPG biosynthesis. In addition, we observed a more pronounced reduction in viral titre following COG6-/- versus COG7-/- (4.3-fold vs. 1.5-fold respectively), and more severe impact on glycosylation in the former (Figure 5d). By lectin staining, we also observed that protein(s) in viruses produced by COG6-/- cells had reduced N-glycosylation, potentially explaining the reduced infectivity of these viruses (Figure 5c). It could be that during a multi-cycle infection in these KO cells, the cumulative effect of less infectious viruses from the first cycles resulted in lower virus titers at the end of a multiple cycles of infection (Figure 4a,b,d).

The COG complex is formed by two lobes, with lobe A containing subunits COG1-4 and lobe B composed of COG5-8 [36]. Each subunit has been found to interact with overlapping yet non-identical subsets of Rab GTPases, golgins, and SNAP receptors (SNAREs) [36], implicating differential outcomes from individual subunit KO. As the COG complex participates in multiple aspects of the Golgi functionality such as intra-cellular trafficking and protein glycosylation, our data do not rule out the possibility that in addition to HS deficiency and impaired N-glycosylation as observed in our study (Figure 4 and Figure 5), COG complex perturbation could result in additional cellular defects that contribute to impaired virus replication. By comparing GLCE KO cells with COG deficient cells, one study observed similar profiles of glycosylation impairment in the two lines but also glycosylation-independent cellular defects in the latter, including fragmented Golgi, abnormal endolysosomes, defective sorting or delayed retrograde trafficking [71]. These defects probably reduced fitness of the COG6 KO cells and VP26-GFP protein levels (Figure 4b), enabling us to identify the COG6 as a candidate gene at the first place through our screen, which was designed to isolate cells with lower GFP levels in infected cells by FACS [49].

Finally, we demonstrated that CRISPRi can be a powerful tool when applied to modulate host gene expression in the context of a viral infection. To mitigate gaps in our knowledge relating to accurate TSS and nucleosome occupancy information for cattle, we developed an easy to assemble multiplex CRISPRi system, CRISPR3i, that utilises three guides simultaneously. Using this approach, we successfully validated eight out of nine candidates obtained from the CRISPRko screen that have been suggested with roles in trafficking or catalyzing the formation of HS precursor molecules (PTAR1, SLC35B2, SLC39A9, UGDH and UGP2) [57,58] or with indirect involvement in protein glycosylation (TM9SF2, TMED2, TMEM165 and UNC50, Figure 7c). The failure to validate UGDH does not necessarily indicate false positivity as the KD threshold required for phenotypic results may not have been reached. Here we used CRISPR3i to target a single gene at a time, but an alternative approach could be to employ guides designed to different genes but expressed from a single vector. It would be relatively simple to further expand this system to deliver a greater number of guides simultaneously.

The full potential CRISPRi can offer to large animal research remains to be explored. RNA interference (RNAi) primarily degrades or blocks the translation of transcripts that are exported to the cytoplasm, leaving those that reside and function in the nucleus largely unaffected. This means that many viral non-coding or host non-coding RNAs cannot be adequately interrogated by RNAi. CRISPRi by contrast can be designed to repress the expression of almost any gene, regardless of the destination of the transcripts. CRISPRi is also an excellent alternative to KO when targeting genes essential for cell fitness or those from large gene families with high sequence similarity between members. Furthermore, the inducible nature of CRISPRi allows the level of KD to be tuned by varying the doxycycline dose (Figure 6e). In the current study we utilised CAGE data from water buffalo [72] to refine our bovine TSS prediction, although less than 30% of these CAGE tags were mapped to the vicinity of annotated promoters on a genome wide level (unpublished data). Ongoing efforts to better annotate a plethora of genomes will undoubtedly contribute to improved CRISPRi design.

## Data Availability

All data generated in this study have been included in the manuscript.

## Supplementary Materials

The following supporting information can be downloaded at: https://www.mdpi.com/article/10.3390/v16020297/s1. the Supplementary Materials file contains Figure S1. STRING analysis of all candidates with suspected roles in cell entry. Figure S2. COG6KO reduced AIHV-1 replication. Figure S3. Comparison of CRISPRi KD efficiency in heterozygote and homozygote dCas9 cell lines.; Table S1. Guide RNA sequences for CRISPRko and CRISPRi. Table S2. qPCR primers for viral genes and for CRISPRi validation. Table S3. Genotypes of CRISPR KO clones. Table S4. oligo sequences used for cloning the CRISPR3i vectors.
