# Supplementary material for: Validation of Candidate Host Cell Entry Factors for Bovine Herpes Virus Type-1 Based on a Genome-Wide CRISPR Knockout Screen
Source: Viruses. 2024 Feb 15;16(2):297. doi: 10.3390/v16020297 (PMC10893506; doi:10.3390/v16020297)
Supplement: Supplementary file 1 [file viruses-16-00297-s001.zip › viruses-2789917-supplementary.pdf]

Validation of Candidate Host Cell Entry Factors for Bovine Herpes Virus Type-1 Based on a Genome-Wide CRISPR Knockout Screen

Wenfang Spring Tan [1,*,†], Enguang Rong [1,†], Inga Dry [1], Simon Lillico [2,3], Andy Law [4], Paul Digard [1], Bruce Whitelaw [2,4] and Robert G. Dalziel [1]

[1] Division of Infection and Immunity , the Roslin Institute, Easter Bush Campus, University of Edinburgh, Edinburgh EH259RG, UK; inga.dry@roslin.ed.ac.uk (I.D.); paul.digard@roslin.ed.ac.uk (P.D.); robert.dalziel@ed.ac.uk (R.G.D.)
[2] Division of Functional Genetics and Development, the Roslin Institute, Easter Bush Campus, University of Edinburgh, Edinburgh EH259RG, UK; simon.lillico@roslin.ed.ac.uk (S.L.); bruce.whitelaw@roslin.ed.ac.uk (B.W.)
[3] Centre for Tropical Livestock Genetics and Health, the Roslin Institute, Easter Bush Campus, University of Edinburgh, Edinburgh EH259RG, UK
[4] Division of Genetics and Genomics, the Roslin Institute, Easter Bush Campus, University of Edinburgh, Edinburgh EH259RG, UK; andy.law@roslin.ed.ac.uk
* Correspondence: wtan2@exseed.ed.ac.uk
† These authors contributed equally to this work.

Keywords: CRISPR/Cas9, BoHV-1, Cell entry, Receptors, Heparan Sulfate, Golgi apparatus, COG complex

Supplementary Materials, Methods and Data

Detailed CRISPR3i cloning protocol

Step1. Digest 10ug of pKLV2-U6gRNA5(BbsI)-PGKpuro2ABFP-W (referred to as PB_3i from now on, Addgene #67974) >=4hrs, gel purify vector and elute with 50ul H2O to obtain linear vector. Alternatively, use uncut PB_3i in Step3 directly but background may be higher if digestion is not complete.

Step2. Assemble the re-annealing reactions below;

Components	Volume (uL)
sgRNA sense + anti-sense oligo (50 µM)	1
10X NEB Buffer 2	0.5
ddH2O	3.5
Total	5

Anneal the oligos in a thermocycler by using the following parameters: 95 °C for 5 min; ramp down to 25 °C at 0.1°C/s, 4°C hold;
Mix 1ul of re-annealed oligos from each g1, g2 and g3 with 97ul nuclease free H20 for a 1:100 dilution;
Proceed with BbsI digest and ligation;

Step 3. Digestions and ligation:
Assemble the reactions in strip tubes below, replace oligo mix with H2O as negative control:

Components	Volume (uL)
PB_3i (~50 ng/ul, linear or circular)	0.25
TOPO_g1tracr_H1(100ng/ul)	0.5
TOPO_g2tracr_mU6(100ng/ul)	0.5
Diluted oligo duplex mixes from above	1
10X CutSmart buffer	1
NEB BbsI(10u/ul)	0.5
Quick T4 ligase(2000u/ul)	0.25
10mM ATP	0.5
20mM DTT	0.25
ddH2O	5.25
Total	10

Incubate reactions in a thermo cycler running the following program: 10 X (37°C, 5 min; 16°C, 10 min); 37°C, 30 min + 65°C, 10 min.

Step 4. Assemble the following plasmid-safe nuclease treatment and incubate reaction at 37°C for 30 min:

Components	Volume (uL)
Reaction from above	10
PlasmidSafe buffer	1.5
H2O	1.75
10mM ATP	1.25
Plasmid-Safe nuclease	0.5
Total	15

Step 5. Transform 2ul of the above reactions into 25 ul of Stbl3 or NEB Stable competent cells and plate the transformed E. Coli onto Carb^{50} or Amp^{100} plates.

Step 6. The second day, pick colonies for bacterial PCR and start 5ml of culture for minipreps using Qiagen miniprep columns and Plasmid plus buffers with endotoxin removal. Elute with 30ul of endotoxin-free, EB, TE or H2O.

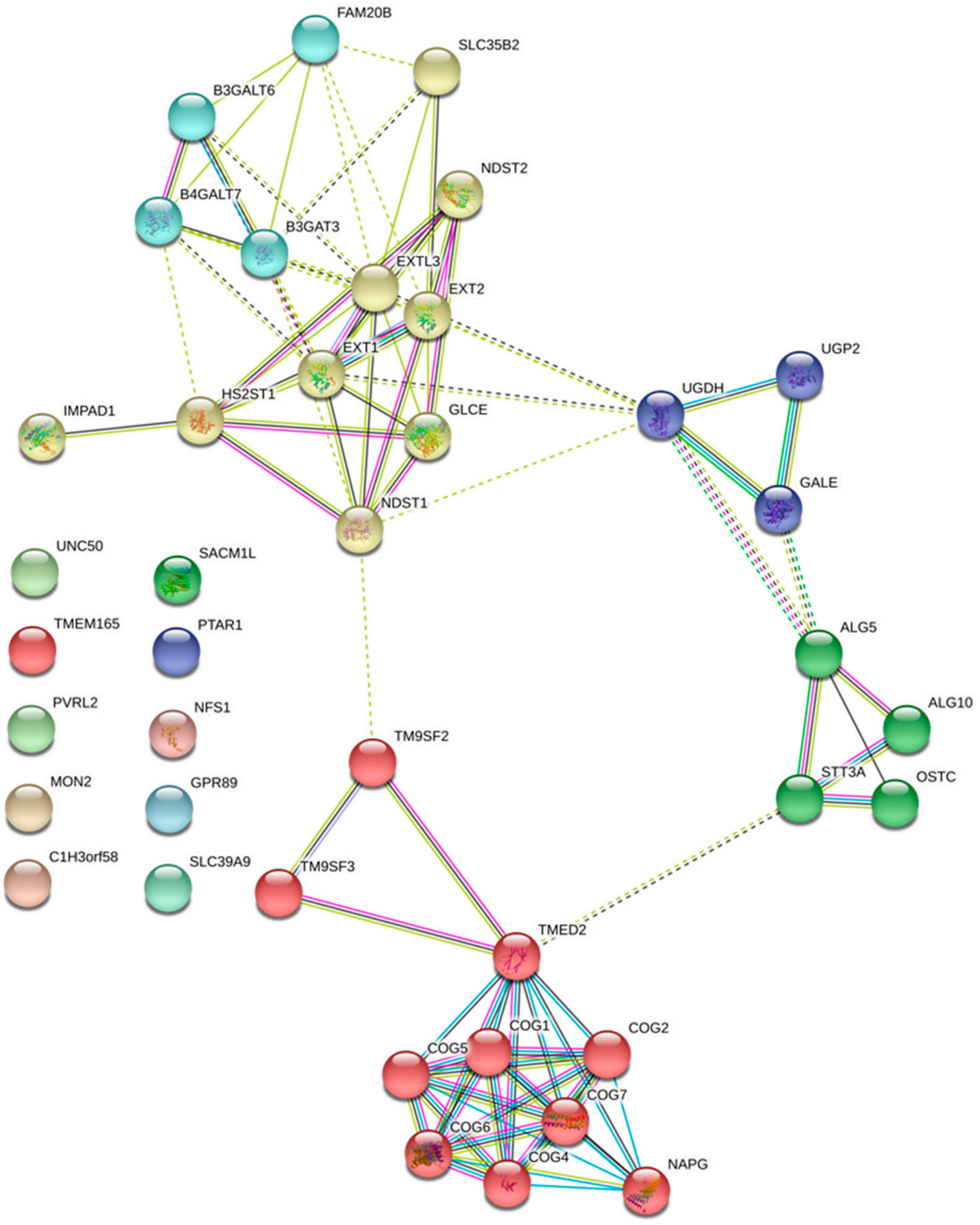

Figure S1. STRING analysis of all candidates with suspected roles in cell entry.

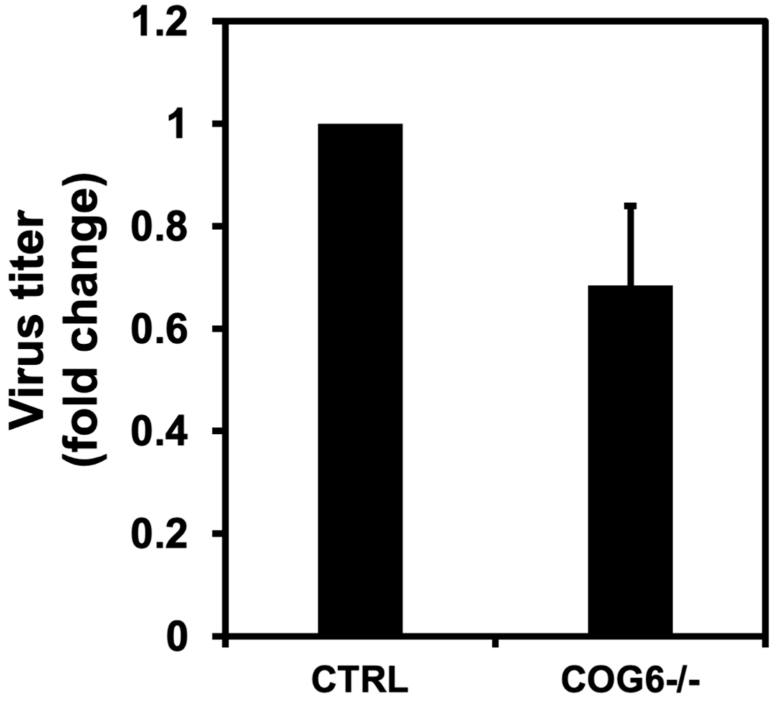

Figure S2. COG6KO reduced AlHV-1 replication. Plaque assay was conducted in Cas9+/+ control cells and COG6 KO cells infected with AlHV-1. Virus titers are presented as relative numbers with that in controls cells set as 1 (n=3, **p=0.016**).

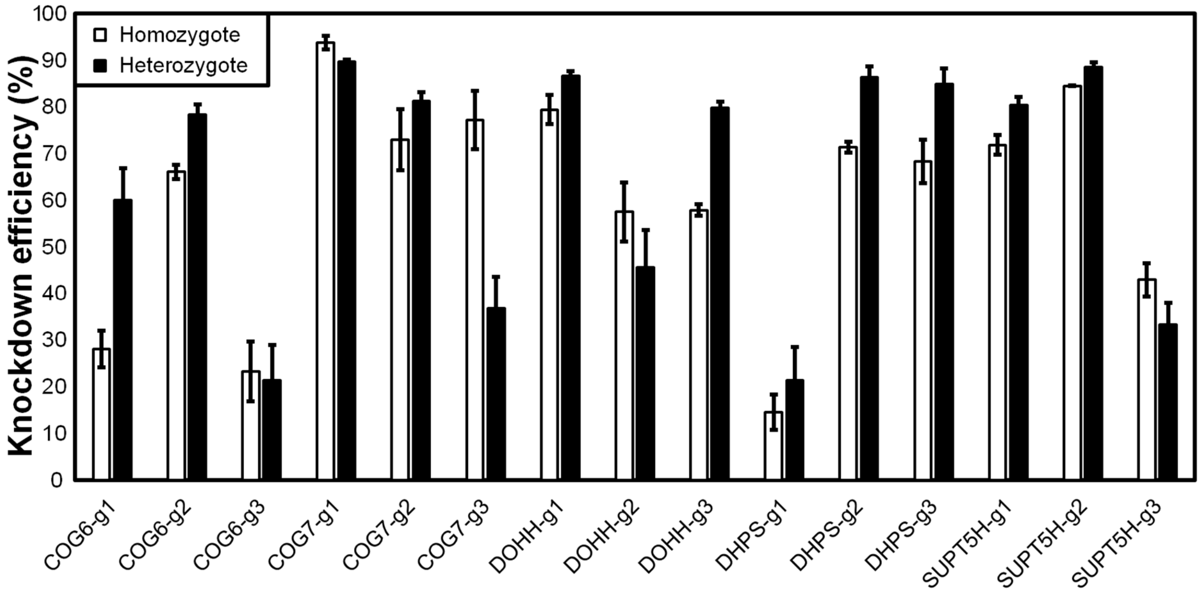

Figure S3. Comparison of CRISPRi KD efficiency in heterozygote and homozygote dCas9 cell lines. KD efficiency was assayed using RT-qPCR using mRNA samples collected from dCas9+/+ or dCas9/wt MDBKs transfected with PiggyBac vector expressing one of the three guides (g1,g2,g3) targeting one of the five genes tested. KD efficiency was calculated by $2^{-\Delta\Delta CT}$ using GAPDH as an internal control.

Table S1. Guide RNA sequences for CRISPRko and CRISPRi

Gene	I.D.	Guide sequence	purpose
**PVR**	NA	GCTCTTGTCACCATTAGGGA	KO
**PVRL2**	NA	GTCGGCGGCGACAATCTCAA	KO
**GLCE**	NA	CAGAGTCAAGTGCATAAGTG	KO
**HST2ST1**	NA	GATGAATGTGTAGCCGAGGG	KO
**COG6**	NA	TTACGAAAGACTTTACCGGT	KO
**COG7**	NA	GTGTTGGTAGAAATCGACC	KO
**COG6**	g1	CGGGTTGCTGGTCTGCGCCG	CRISPRi
**COG6**	g2	GGACAACGACAAGGTGAGCG	CRISPRi
**COG6**	g3	AGTCGTAGTGCCCGCGACTG	CRISPRi
**COG7**	g1	CGTGAAGGAGTGGATCAACG	CRISPRi
**COG7**	g2	CCAAGAGGTGAACCACGCCG	CRISPRi
**COG7**	g3	ACATCAGCGCGCTGATCCAG	CRISPRi
**DOHH**	g1	AGTGAAGAATCAGCGCGCGG	CRISPRi
**DOHH**	g2	TCCGATCTGGTAGCTCACTG	CRISPRi
**DOHH**	g3	TGTGAAGGATCAGGTACTGG	CRISPRi
**DHPS**	g1	CGAACTGTGCTTCAGCACGG	CRISPRi
**DHPS**	g2	GTGCGCGATAGTCCACGCCA	CRISPRi
**DHPS**	g3	GGTACAGCAAGTCAACGCCA	CRISPRi
**SUPT5H**	g1	CGAGGACAGCAACTTCTCCG	CRISPRi
**SUPT5H**	g2	AGGCTAAAGAGTCGCAAGAG	CRISPRi
**SUPT5H**	g3	CCGAACTGGGAGTTGCGCTG	CRISPRi

Table S2. qPCR primers for viral genes and for CRISPRi validation

Gene	Forward	Reverse
**UL23**	CTCTGCTACCCCTTCGCCCGCTACT	AGGGTGCACACGACGAGGTTGGC
**COG6**	GAGTTTGGAACAGCCAGAAGAAGTA	CGAGGTGTTCTTTCTCAGAAGCAGT
**COG7**	TGCTCCTCATTCCCAAGATGGATAG	TTCAGGGGGAGGGACATGATATACT
**DOHH**	TGCTCAAGCATGAGCTGGCCTACT	CCACCTCAACAACAGGGTCAGTAGA
**DHPS**	GTGGAGGAGGATTTCATCAAGTGCT	CACTTCACACCCTCTGTGTTCTGCT
**SUPT5H**	ATTGCCTACCAGTTCACAGACACGC	ATCCGTCATCTCCTTGATAGGCACC

Table S3. Genotypes of CRISPR KO clones

Gene	Clone I.D.	Genotype
PVR	23	-50/-50
PVR	25	-2/-11
PVR	29	-8/-8
PVR	32	-5/-33
PVRL2	A4	-1/+1
PVRL2	A11	-4/+1
PVRL2	C11	-4/+1
PVRL2	D5	-19/-19
GLCE	A1	-13/+1
GLCE	A7	+1/+1
GLCE	B2	-8/-8
GLCE	B8	+1/+1
HST2ST1	A5	+1/+1
HST2ST1	C1	-10/-11
HST2ST1	C2	-1/+1
HST2ST1	C4	+1/+1
COG6	A4	-13/-13
COG6	A6	-1/+1
COG6	A9	-20/-20
COG6	C3	-152/-152
COG6	C6	+1/+1
COG7	A3	-32/-32
COG7	C5	-37/-37
COG7	F3	-13/-13
COG7	F7	-5/-5

Table S4. Oligos used for CRISPRi cloning.

CRISPRi sequences are underscored	
**Sequence Name**	Sequence
**3iG1_PTAR1::45886631+:NC_037335.1**	CAC CGA TAC CTA GCT TCA GGA CAC GTT
**3iG1_PTAR1::45886631-:NC_037335.1**	CTT AAA CGT GTC CTG AAG CTA GGT ATC
**3iG2_PTAR1::45886607+:NC_037335.1**	ATC TGA ACC AGG ACT ATG GGA CTC GTT TAA
**3iG2_PTAR1::45886607-:NC_037335.1**	GCT CTT AAA CGA GTC CCA TAG TCC TGG TTC
**3iG3_PTAR1::45886540+:NC_037335.1**	TGT TGG GAG CTT GTT GTG GAC ATA
**3iG3_PTAR1::45886540-:NC_037335.1**	AAA CTA TGT CCA CAA CAA GCT CCC
**3iG1_SLC35B2::17762949+:NC_037350.1**	CAC CGG GAT CGG GAG AGA AGT CGC GTT
**3iG1_SLC35B2::17762949-:NC_037350.1**	CTT AAA CGC GAC TTC TCT CCC GAT CCC
**3iG2_SLC35B2::17763261+:NC_037350.1**	ATC TGT TGC ACT TCC GCT GGC CGG GTT TAA
**3iG2_SLC35B2::17763261-:NC_037350.1**	GCT CTT AAA CCC GGC CAG CGG AAG TGC AAC
**3iG3_SLC35B2::17762887+:NC_037350.1**	TGT TGA GGG CGC CGC GCC TCC AAC
**3iG3_SLC35B2::17762887-:NC_037350.1**	AAA CGT TGG AGG CGC GGC GCC CTC
**3iG1_SLC39A9::81185607+:NC_037337.1**	CAC CGC TCC TCC AAG TCT AGA GTG GTT
**3iG1_SLC39A9::81185607-:NC_037337.1**	CTT AAA CCA CTC TAG ACT TGG AGG AGC
**3iG2_SLC39A9::81185495+:NC_037337.1**	ATC TGC ATG TTA CTC CAC TCC GAG GTT TAA
**3iG2_SLC39A9::81185495-:NC_037337.1**	GCT CTT AAA CCT CGG AGT GGA GTA ACA TGC
**3iG3_SLC39A9::81185759+:NC_037337.1**	TGT TGC CAC AAG CAA TGT TCC CGA
**3iG3_SLC39A9::81185759-:NC_037337.1**	AAA CTC GGG AAC ATT GCT TGT GGC
**3iG1_TM9SF2::76343648+:NC_037339.1**	CAC CGG CGC CCG TCA ACT TCT GCG GTT
**3iG1_TM9SF2::76343648-:NC_037339.1**	CTT AAA CCG CAG AAG TTG ACG GGC GCC
**3iG2_TM9SF2::76343397+:NC_037339.1**	ATC TGA CCG GAA CTC TCC GTC GCG GTT TAA
**3iG2_TM9SF2::76343397-:NC_037339.1**	GCT CTT AAA CCG CGA CGG AGA GTT CCG GTC
**3iG3_TM9SF2::76343686+:NC_037339.1**	TGT TGG CGC CCA CCT TGC ACT CGT
**3iG3_TM9SF2::76343686-:NC_037339.1**	AAA CAC GAG TGC AAG GTG GGC GCC
**3iG1_TMED2::52090946+:NC_037344.1**	CAC CGC AGC TAT CCG AGT CCT GGG GTT
**3iG1_TMED2::52090946-:NC_037344.1**	CTT AAA CCC CAG GAC TCG GAT AGC TGC
**3iG2_TMED2::52091065+:NC_037344.1**	ATC TGT TCC TGG ACA TCG ACG TGG GTT TAA
**3iG2_TMED2::52091065-:NC_037344.1**	GCT CTT AAA CCC ACG TCG ATG TCC AGG AAC
**3iG3_TMED2::52091089+:NC_037344.1**	TGT TGC TCG GCC ACC TCA AAG ATG
**3iG3_TMED2::52091089-:NC_037344.1**	AAA CCA TCT TTG AGG TGG CCG AGC
**3iG1_TMEM165::70838793+:NC_037333.1**	CAC CGC AAC AGA GGA CGT GAG ACA GTT
**3iG1_TMEM165::70838793-:NC_037333.1**	CTT AAA CTG TCT CAC GTC CTC TGT TGC

**3iG2_TMEM165::70839078+:NC_037333.1**	ATC TGT GGC GGT TCC TTG TTC CGG GTT TAA
**3iG2_TMEM165::70839078-:NC_037333.1**	GCT CTT AAA CCC GGA ACA AGG AAC CGC CAC
**3iG3_TMEM165::70838985+:NC_037333.1**	TGT TGC AGC CCG GGC GCA GAT GCG
**3iG3_TMEM165::70838985-:NC_037333.1**	AAA CCG CAT CTG CGC CCG GGC TGC
**3iG1_TMEM41B::43311362+:NC_037342.1**	CAC CGG GAT CCT TAC AAC ACC CAG GTT
**3iG1_TMEM41B::43311362-:NC_037342.1**	CTT AAA CCT GGG TGT TGT AAG GAT CCC
**3iG2_TMEM41B::43311459+:NC_037342.1**	ATC TGG TCG AAA GAT CGC AAA CGG GTT TAA
**3iG2_TMEM41B::43311459-:NC_037342.1**	GCT CTT AAA CCC GTT TGC GAT CTT TCG ACC
**3iG3_TMEM41B::43311568+:NC_037342.1**	TGT TGA GAC CTG GGA ACC AGC GCG
**3iG3_TMEM41B::43311568-:NC_037342.1**	AAA CCG CGC TGG TTC CCA GGT CTC
**3iG1_UGDH::58620020+:NC_037333.1**	CAC CGT AGA AGT GGT TGC AGC ACT GTT
**3iG1_UGDH::58620020-:NC_037333.1**	CTT AAA CAG TGC TGC AAC CAC TTC TAC
**3iG2_UGDH::58620252+:NC_037333.1**	ATC TGG GAG CAG GGA TGC AAC CGA GTT TAA
**3iG2_UGDH::58620252-:NC_037333.1**	GCT CTT AAA CTC GGT TGC ATC CCT GCT CCC
**3iG3_UGDH::58620127+:NC_037333.1**	TGT TGG CAT CCC GAG AAG GGT GTG
**3iG3_UGDH::58620127-:NC_037333.1**	AAA CCA CAC CCT TCT CGG GAT GCC
**3iG1_UGP2::62288834+:NC_037338.1**	CAC CGA AGA TAG TTA TGT ACT CGG GTT
**3iG1_UGP2::62288834-:NC_037338.1**	CTT AAA CCC GAG TAC ATA ACT ATC TTC
**3iG2_UGP2::62288971+:NC_037338.1**	ATC TGT TCT CAG CCG CAG AAC GCG GTT TAA
**3iG2_UGP2::62288971-:NC_037338.1**	GCT CTT AAA CCG CGT TCT GCG GCT GAG AAC
**3iG3_UGP2::62288921+:NC_037338.1**	TGT TGT TGC TAA AGT ATC TGT GGA
**3iG3_UGP2::62288921-:NC_037338.1**	AAA CTC CAC AGA TAC TTT AGC AAC
**3iG1_UNC50::3824412+:NC_037338.1**	CAC CGC TGC GTG CTC AAG GTA GCG GTT
**3iG1_UNC50::3824412-:NC_037338.1**	CTT AAA CCG CTA CCT TGA GCA CGC AGC
**3iG2_UNC50::3824300+:NC_037338.1**	ATC TGC CTA AGA CCG CGA ACT CGG GTT TAA
**3iG2_UNC50::3824300-:NC_037338.1**	GCT CTT AAA CCC GAG TTC GCG GTC TTA GGC
**3iG3_UNC50::3824390+:NC_037338.1**	TGT TGC CTG GCG CTC GGA TGA CCG
**3iG3_UNC50::3824390-:NC_037338.1**	AAA CCG GTC ATC CGA GCG CCA GGC
**3iG1_nc_guide74+**	CAC CGC GCG GCG TCC ATA TCA CGT GTT
**3iG1_nc_guide74_**	CTT AAA CAC GTG ATA TGG ACG CCG CGC
**3iG2_nc_guide95+**	ATC TGC GTA CTC GTA TAC TAC TCG GTT TAA
**3iG2_nc_guide95-**	GCT CTT AAA CCG AGT AGT ATA CGA GTA CGC
**3iG3_nc_guide606+**	TGT TGT GAC GCG TGG TCG AAC CGC
**3iG3_nc_guide606-**	AAA CGC GGT TCG ACC ACG CGT CAC
